# Gait in children with achondroplasia – a cross-sectional study on joint kinematics and kinetics

**DOI:** 10.1186/s12891-022-05343-4

**Published:** 2022-04-28

**Authors:** Eva W. Broström, Lotte Antonissen, Johan von Heideken, Anna-Clara Esbjörnsson, Lars Hagenäs, Josefine E. Naili

**Affiliations:** 1 Department of Women’s and Children’s Health, Karolinska Institutet, Motoriklab, Q2:07, Karolinska University Hospital, 171 76 Stockholm, Sweden; 2 Faculty of Movement and Rehabilitation Sciences, Katholieke Universiteit Leuven, Tervuursevest 101, 3001 Leuven, Belgium; 3 Department of Clinical Sciences Lund, Orthopaedics, Lund University, Skane University Hospital, 221 85 Lund, Sweden

**Keywords:** Achondroplasia, Children, Walking, Gait analysis, Kinematics, Kinetics

## Abstract

**Background:**

Children with achondroplasia have extreme short stature due to short limbs, as well as several other clinical features that may affect their gait. The purpose of this cross-sectional study was to provide a detailed description of gait in children with achondroplasia compared to age-matched controls.

**Methods:**

Between the years 2007 and 2010, 16 children with achondroplasia [mean age 9.6 years (range 5–16; six female)] with no previous history of orthopaedic lower limb surgery and 19 age-matched controls conducted three-dimensional (3D) gait analysis at one occasion. The gait analysis rendered pelvis and lower limb joint kinematics and kinetics, and time and distance data. Descriptive statistics, independent samples t-tests, and Fisher’s exact test were used to describe the cohort including gait data and participant characteristics.

**Results:**

Children with achondroplasia had kinematic gait pattern deviations in all three planes, especially in the sagittal plane, when compared to the control group. Peak anterior pelvic tilt and peak ankle dorsiflexion were found to be increased. Increased knee flexion was noted at initial contact and again at terminal stance. During stance, children with achondroplasia had a higher peak hip abduction angle and a higher peak knee varus angle in the frontal plane. In the sagittal plane, kinetic gait pattern deviations were found at the hip, knee, and ankle, consistent with a flexion pattern. Compared to the control group, children with achondroplasia walked with reduced walking speed and step length, and increased cadence. There was no difference in walking speed when leg length was taken into account. Normalised step length and normalised cadence, on the other hand, were found to be increased in children with achondroplasia.

**Conclusions:**

The observed gait characteristics in children with achondroplasia are related to anatomical attributes and strategies to increase step length, and hence walking speed.

## Background

Achondroplasia, a metaphyseal skeletal dysplasia, is a genetic condition with a prevalence in Europe of 3.7 per 100,000 births [1]. The condition is associated with delayed gross motor development and characterized by extreme short stature [2]. The disproportional stature is mainly dependent on short limbs in contrast to sitting height being almost average for the background population [3]. The shorter legs affect the time and distance parameters of walking, including reduced stride length and lower walking speed [4, 5]. Other common clinical features that may affect walking function and gait patterns are reduced lower limb muscle strength, relatively longer foot-to-leg ratio, large head size, limited elbow extension, thoracolumbar kyphosis, lumbar hyperlordosis and broad pelvis. Additional functional features include increased hip abduction, often in combination with hip flexion contractures, hypermobile knees depending on ligament laxity causing medio-lateral knee instability with lateral thrust in standing and genu recurvatum [3]. With time bowing of tibia often develops, caused by dynamic instability of the knee as well as bone deformities i.e., varus of the tibia, internal tibial torsion, and a distal overgrow of fibula in relation to the tibia [3, 4, 6, 7]. Children with achondroplasia may also experience musculoskeletal pain and neurological impairment affecting their gait [3, 8].

A recent qualitative study concluded that a majority children and adolescents with achondroplasia report difficulty walking long distances or for long periods of time, and that pain (back, joint, and leg) was the most commonly mentioned symptom [9]. Few studies have analysed the impact of the musculoskeletal differences on gait patterns in children [5] and adults [10, 11] with achondroplasia compared to age-matched controls. Kinetic analysis, including joint moments, of gait in children with achondroplasia has previously, to our knowledge, only been addressed in one study without a control group [4], and two conference abstracts [12, 13], out of which one was based on a single subject [12].

A full description of gait patterns in children with achondroplasia, including lower limb joint kinematics and kinetics, and time and distance parameters, would contribute to the currently scarce body of literature with relatively small study populations. Furthermore, detailed descriptions of gait patterns may provide deepened knowledge of achondroplasia specific gait characteristics, which in turn may be interpreted in relation to known physical symptoms and pain, and thereby inform health care and guide rehabilitation.

This cross-sectional study aimed to evaluate gait patterns, as detailed above, in children with achondroplasia and compare to a group of age-matched controls. It was hypothesised that children with achondroplasia would display gait deviations with a pattern of increased anterior pelvic tilt and flexion in lower extremity joints, accompanied by deviating joint kinetics that (at least in part) depends on the special skeletal configuration.

## Methods

The study was performed in accordance with the Declarations of Helsinki [14]. The STROBE guidelines were followed in the development and reporting of this study [15].

### Study participants and clinical measurements

Between the years 2007 and 2010, three-dimensional (3D) gait analysis was performed in 16 children diagnosed with achondroplasia. Inclusion criteria were achondroplasia according to clinical and radiological criteria [16], no previous history of orthopaedic lower limb surgery, and the ability to walk 10 m repeatedly without assistance of a walking aid. Nineteen age-matched children were recruited amongst acquaintances and friends, and constituted the control group. Height and weight of all included participants were measured using calibrated scales. Weight was measured to the neatest 100 g. Among children with achondroplasia, body proportions were evaluated as sitting height, sitting height ratio (SHR; sitting height/total height) expressed in percent, and sitting height expressed in standard deviation scores (SH SDS) [17, 18]. Leg length was measured as the distance between the anterior superior iliac spine and the medial malleolus of the ankle, with the child in a supine position. All clinical measurements were performed by an experienced physiotherapist (EWB).

### Gait analysis

3D gait analysis was conducted in a Motion Analysis Laboratory, equipped with an eight-camera system, Vicon MX (sampling rate 100 Hz) (VICON, Oxford, UK) and two staggered Kistler force plates embedded in the floor (Kistler, Winterthur, Switzerland), at Astrid Lindgren Children’s Hospital, Karolinska University Hospital, Stockholm, Sweden, a tertiary children’s hospital. Reflective markers were placed on anatomical landmarks according to the conventional biomechanical Plug-In-Gait model [19]. Kinematic, kinetic, and time and distance data were collected simultaneously while children walked barefoot at a self-selected walking speed [20]. In four children with achondroplasia and one child in the control group, no clean force plate strikes were obtained due to short step length. For these children, only kinematic and time- and distance data were included in the analysis.

### Data analysis

Gait data was processed using Vicon Nexus software (2.9.1), and raw motion capture data was filtered using a Woltring filter. The kinematic data of interest included pelvis and hip kinematics in the sagittal, frontal and transverse plane, knee kinematics in the sagittal and frontal plane, ankle kinematics in the sagittal plane, and foot progression (the foot relative to the global coordinate system of the laboratory). Kinetics were expressed by internal moments and normalized by bodyweight, and included hip and knee moments in the sagittal and frontal plane, and ankle moments in the sagittal plane. Three gait cycles, with clean force plate strikes, consistent walking speed, and good marker quality data, were analysed for each participant. For the control group, data of the left side was arbitrarily chosen and included in the analysis. For children with achondroplasia, efforts were made to include the side of the body with the most available kinetic data in the analysis, either the left *or* the right side, whichever side had at least three strides with clean force plate strikes. For each child, data derived from the included gait cycles were averaged. Time normalized kinematic and kinetic waveforms were plotted using MATLAB (MATLAB and Statistical Toolbox Release R2019b, The MathWorks, Inc., Natick, Massachusetts, US). Self-selected walking speed (velocity from heel strike to subsequent heel strike), and step cadence (steps/minute) were normalised by leg length and gravity, and step length by leg length as described by Hof [21].

### Statistical analysis

Descriptive statistics were used to describe the cohort. Mean values with standard deviations were calculated for normally distributed continuous data (participant characteristics, gait kinematics, gait kinetics, time and distance parameters), and percentages for categorical data (gender). Normal distribution of data was assessed using Shapiro-Wilk’s test and Q-Q plots. Independent samples t-tests were used to evaluate differences in participant characteristics, kinematic, kinetic and time and distance parameters between children with achondroplasia and the control group. Fisher’s exact test was used to assess differences in proportions of boys and girls between children with achondroplasia and controls. A statistical significance level was set at ɑ = 0.05. Statistical analyses were performed using IBM SPSS Statistics version 28 (Chicago, IL).

## Results

### Participant characteristics

Sixteen children with achondroplasia [mean age 9.6 years (range 5–16; six female)] and 19 age-matched control children were included in the study. Children with achondroplasia were shorter and had shorter legs, and a mean sitting height ratio of 67% (Table 1).

**Table 1:** Participant characteristics of included children

	Achondroplasia group (***n*** = 16)	Control group (***n*** = 19)	***p***-value
**Characteristics**	Mean (SD)		
Age, years	9.6 (3.7)	10.7 (2.3)	0.317
Female, n (%)	6 (38)	11 (58)	0.315
Leg length, cm	43 (8)	78 (10)	**0.000**
Height, cm	105 (15)	143 (15)	**0.000**
Bodyweight, kg	29.4 (14.2)	36.3 (10.4)	0.105
SH, cm	70 (9)	N/A	
SHR, %	67 (1.4)	N/A	
SH SDS	−0.85 (1.16)	N/A	

*SD* Standard Deviation, *SH* Sitting Height, *SHR* Sitting Height Ratio, *SH SDS* Sitting Height expressed in Standard Deviation Scores, *N/A* Not Applicable

### Gait kinematics

**Fig. 1:**
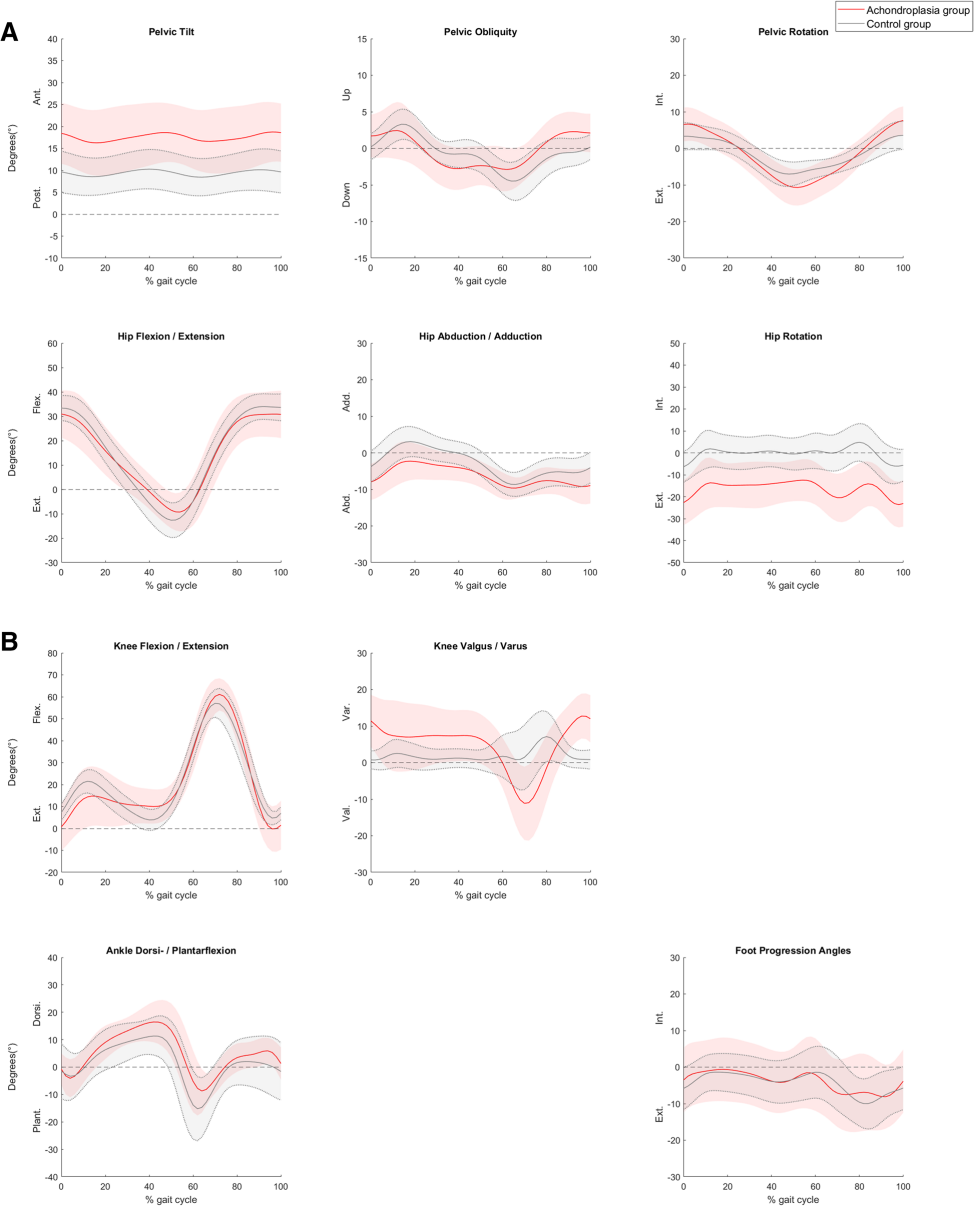
Joint kinematics of **A**) the pelvis and hip joint in the sagittal, frontal, and transverse plane **B**) the knee joint in the sagittal and frontal plane, ankle joint in the sagittal plane, and foot progression (the foot relative to the global coordinate system of the laboratory) during level gait at self-selected speed in a group of children with achondroplasia (*n* = 16) and a control group (*n* = 19). The solid red line represents the group mean of children with achondroplasia, and the solid grey line represents the group mean of the control group. The corresponding shaded areas represent the mean ± 1 standard deviation of each group, respectively

Children with achondroplasia displayed kinematic gait pattern deviations in all three planes as compared to children in the control group, particularly pronounced in the sagittal plane (Fig. 1A). Increased peak anterior pelvic tilt, and peak ankle dorsiflexion were observed (Table 2). In the knee, increased knee flexion was observed at initial contact, and also during terminal stance (Table 2, Fig. 1B). In the frontal plane, children with achondroplasia displayed increased peak hip abduction angle, and increased peak knee varus angle during stance (Table 2). In the transverse plane, increased range of motion was observed in the pelvis, and increased peak external rotation was observed in the hips (Table 2, Fig. 1A).

**Table 2:** Joint kinematics and kinetics during level gait at self-selected walking speed in a group of children with achondroplasia and in a group of age-matched controls

Joint kinematics (degrees)	Achondroplasia group (***n*** = 16)	Control group (***n*** = 19)	***p***-value
**Pelvis**	Mean (SD)		
Peak anterior pelvic tilt	20.0 (7.1)	11.0 (4.4)	**0.000**
Pelvic obliquity range	7.4 (2.1)	8.0 (3.3)	0.486
Pelvic rotation range	19.6 (6.4)	12.2 (4.8)	**0.000**
**Hip**			
Peak hip flexion (+)	33.2 (9.8)	34.9 (5.5)	0.508
Peak hip extension (−)	−9.8 (7.8)	−12.9 (7.2)	0.230
Peak hip adduction (+)	−1.4 (4.9)	3.3 (4.1)	**0.004**
Peak hip external rotation (−)	− 25.8 (9.9)	−7.9 (7.5)	**0.000**
**Knee**			
Knee at initial contact	0.7 (10.7)	7.3 (3.7)	**0.018**
Knee at loading response (10% of gait cycle)	11.7 (13.4)	19.4 (4.7)	**0.025**
Knee at terminal stance (40% of gait cycle)	10.3 (8.0)	4.3 (4.9)	**0.011**
Peak knee varus in stance	12.5 (7.1)	4.1 (3.8)	**0.000**
Peak knee flexion in swing	62.4 (7.3)	57.8 (6.0)	**0.049**
**Ankle**			
Peak ankle dorsiflexion (+)	18.9 (4.7)	12.6 (6.8)	**0.004**
Peak ankle plantarflexion (−)	−11.4 (6.9)	−15.9 (12.1)	0.193
Peak external (−) foot progression	− 12.3 (9.9)	−11.5 (5.9)	0.762
**Kinetics expressed by internal moments (Nm/bodyweight)**	**Achondroplasia group (*n*** **= 12)**	**Control group (*n*** **= 18)**	***p*-value**
**Hip moments**			
Peak hip extension moment	0.44 (0.16)	0.84 (0.26)	**0.000**
Peak hip flexion moment	−0.87 (0.25)	−0.69 (0.20)	**0.033**
Peak hip abduction moment	0.46 (0.14)	0.60 (0.15)	**0.018**
**Knee moments**			
Peak knee flexion moment	−0.20 (0.07)	−0.36 (0.12)	**0.000**
Peak knee extension moment	0.44 (0.21)	0.66 (0.25)	**0.016**
Peak knee valgus moment	0.45 (0.21)	0.39 (0.10)	0.390
**Ankle moments**			
Peak ankle plantarflexion moment	0.90 (0.19)	1.31 (0.20)	**0.000**

*SD* Standard Deviation

### Gait kinetics

**Fig. 2:**
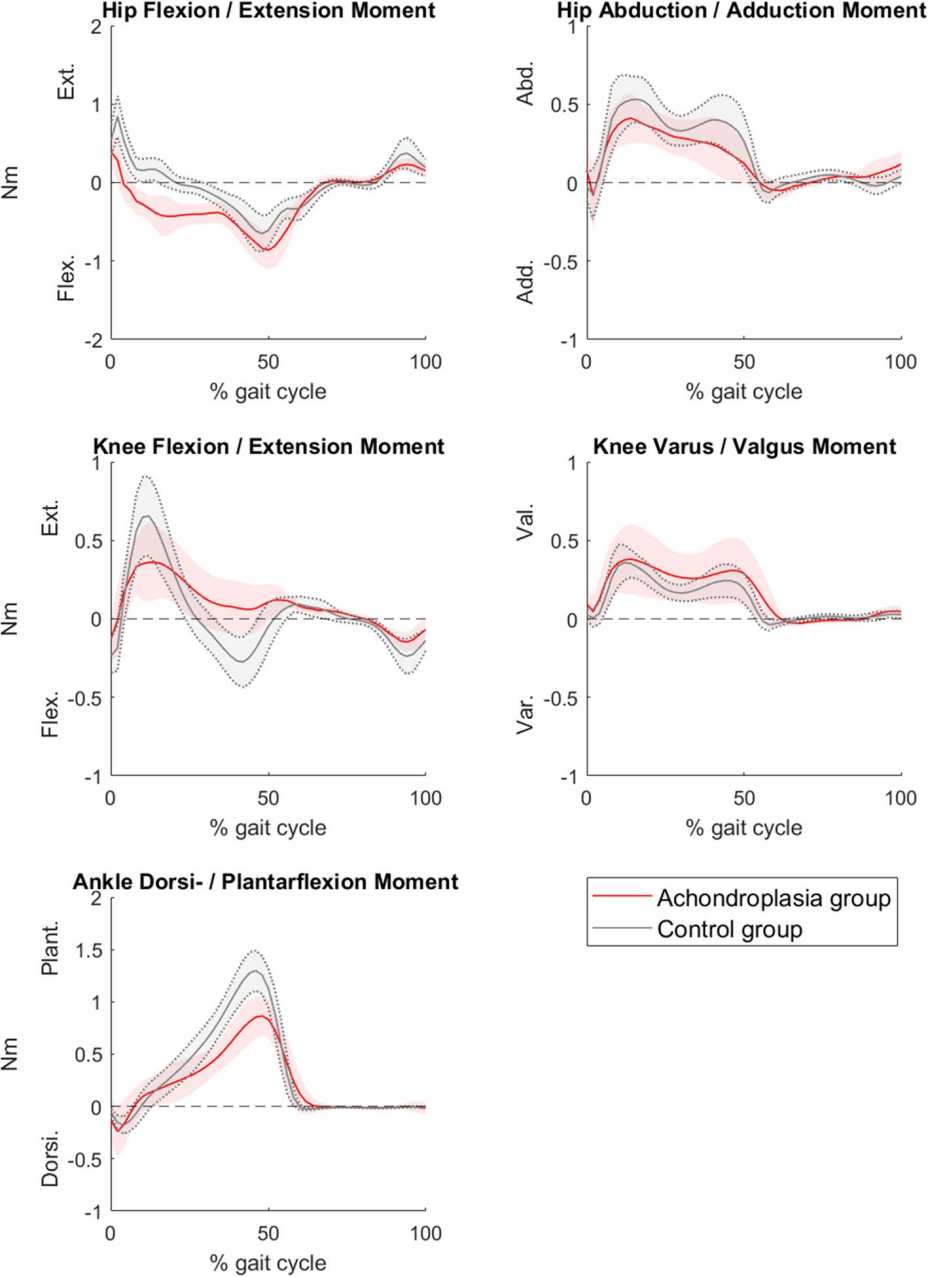
Joint kinetics of the hip, knee, and ankle joints in the sagittal plane, and hip and knee joints in the frontal plane during level gait at self-selected speed in a group of children with achondroplasia (*n* = 12) and a control group (*n* = 18). Joint kinetics are expressed by internal moments and normalized by bodyweight. The solid red line represents the group mean of children with achondroplasia, and the solid grey line represents the group mean of the control group. The corresponding shaded areas represent the mean ± 1 standard deviation of each group, respectively

Children with achondroplasia displayed kinetic gait pattern deviations at the hip, knee and ankle in the sagittal plane, consistent with a flexion pattern (Fig. 2). In the frontal plane, a reduced peak hip abduction moment was observed in children with achondroplasia (Table 2).

### Time and distance parameters

Children with achondroplasia walked with reduced speed and step length, and increased cadence compared to children in the control group (Table 3). When adjusted for leg length, no differences in walking speed remained. However, normalised step length and normalised cadence were found to be increased in children with achondroplasia (Table 3).

**Table 3:** Time and distance parameters during level gait at self-selected walking speed in a group of children with achondroplasia and in a group of age-matched children

	Achondroplasia group (***n*** = 16)	Control group (***n*** = 19)	***p***-value
**Time and distance parameters**	Mean (SD)		
Walking speed, m/s	0.98 (0.20)	1.32 (0.15)	**0.000**
Step length, m	0.38 (0.06)	0.61 (0.08)	**0.000**
Cadence, step/min	152.8 (21.0)	128.3 (14.5)	**0.000**
Normalised walking speed	0.49 (0.10)	0.48 (0.06)	0.977
Normalised^a^ cadence	19.1 (2.8)	12.9 (1.6)	**0.000**
Normalised^b^ step length	0.90 (0.13)	0.79 (0.09)	**0.004**

*SD* Standard Deviation

^a^Normalised to gravity and leg length

^b^Normalised to leg length

## Discussion

This cross-sectional study aimed to provide a detailed description of gait in children with achondroplasia as compared to age-matched controls. In line with the pre-formulated hypothesis, children with achondroplasia walked with a flexion pattern, observed by increased anterior pelvic tilt and flexion in the lower extremity joints. Correspondingly, reduced hip and knee extension moments, and increased hip and knee flexion moments were observed. In addition, lower walking speed, reduced step length, and increased step cadence were found as compared to the control group. These findings were mainly in accordance with previous studies investigating gait deviations in children [4, 5] and adults [10] with achondroplasia.

In accordance with the present study, previous literature reported that children with achondroplasia walk with increased anterior pelvic tilt. The increased pelvic tilt, observed throughout the entire gait cycle, may be related to lumbar hyper lordosis, commonly described in children with achondroplasia, but also to muscular tightness in hip flexors, and abdominal muscle weakness [3, 22]. At initial contact and during loading response, increased knee flexion was observed, which may be secondary to the anterior pelvic tilt. A recently published retrospective kinematic analysis of gait in eight children (4–17 years old) with achondroplasia reported increased knee flexion throughout the entire stance phase of gait [5]. This is in contrast to a study by Inan et al., evaluating gait kinematics and kinetics in 13 children (3–17 years old) with achondroplasia, who reported no alterations in sagittal plane knee kinematics [4]. In the present study, children with achondroplasia displayed increased peak dorsiflexion in stance, and reduced peak plantarflexion during push-off. The increased peak dorsiflexion during late stance corroborate the findings of Kiernan [5], and Sims et al. who evaluated gait in 10 adults (18–27 years old) with achondroplasia [10]. Furthermore, Kiernan and Sims et al. reported increased dorsiflexion in swing, and suggested it to be the result of a relatively longer foot-to-leg ratio.

In the frontal plane, increased hip abduction was observed in children with achondroplasia, and there are several possible reasons for this. Anatomically, the pelvic is different in children with achondroplasia, with a wider and more horizontal acetabulum [3]. The bowing of the legs may also contribute to increased hip abduction, as well as to the increased knee varus found during stance [3]. Furthermore, the increased hip abduction mobility [3], in combination with hip flexor tightness, may also impact frontal plane hip kinematics with increased abduction during gait.

In agreement with findings of previous literature, increased range of motion in the pelvis in the transverse plane was observed [4, 5]. The increased pelvic rotation in early stance and late swing, and increase in external hip rotation may represent a strategy to increase step length and thereby walking speed. To the best of our knowledge, only one previous study [4], and two conference abstracts [12, 13] have reported data on kinetic gait patterns in children with achondroplasia. There is an obvious challenge in collecting data and obtaining clean force plate strikes in small children with variable gait patterns, and individuals with short step length. In the present study, kinetic data from 12 children with achondroplasia demonstrated reduced peak hip and knee extension moments, and increased peak hip and knee flexion moments, coherent with the anteriorly tilted pelvis and flexed hip and knee joints. In the frontal plane, the peak hip abduction moment was found to be reduced compared to children in the control group. In contrast to Inan et al. [4], no increase in peak knee valgus moment was found. It is possible that analysis of the positive valgus moment impulse (integrating the positive section of the curve between heel strike and toe-off) [23], instead of analysing peak moment, could have revealed a difference in knee loading between groups. In the ankle joint, a reduced peak plantar flexion moment was observed. This may relate to the previously discussed relatively longer foot-to-leg ratio [5, 11], where the longer foot leads to increased dorsiflexion during stance and a delayed, less effective push-off.

Children with achondroplasia displayed lower walking speed, reduced step length, and increased cadence. When adjusted for leg length, no difference in walking speed remained between groups, which was in agreement with the findings of Sims et al. [11]. However, normalised step length and cadence were found to be increased in children with achondroplasia, suggesting two strategies to compensate for reduced walking speed related to shorter leg length.

In a recent qualitative study, children and adolescents with achondroplasia were asked to describe physical symptoms and experienced complications [9]. Pain was the most often mentioned symptom, with back pain, joint pain, and leg pain the most common locations and types of pain [9]. The majority of children and adolescents (75%) reported difficulty walking long distances or for long periods of time [9]. Furthermore, children and adolescents also discussed tiring easily and experiencing low stamina. In a wider clinical perspective, and an attempt to interpret findings of the present study in relation to current literature, the observed flexion pattern during gait, and increased hip and knee flexion moments, may contribute to back pain, joint pain and leg pain. The findings of increased cadence and normalised step length during gait could be contributing factors to the reported difficulty walking long distances and the experienced low stamina.

This study holds several limitations that need to be acknowledged. First, we had no a priori power analysis for this specific study. Inclusion of children with achondroplasia was carried out in a pragmatic manner over the course of several years. Among the included children with achondroplasia there was a large age range, which may impact the results as these children were in different phases of growth and development of muscle strength. The cross-sectional study design does not lend itself to determination of cause and effect, but rather offers a detailed baseline description of gait characteristics in children with achondroplasia. As previously described, the data collection took place some years ago (between the years 2007–2010), which raises questions whether the data is superseded. In terms of the gait laboratory equipment, we anticipate we would have had better visibility of markers had the data been collected today, depending on newer and increased number of cameras with improved precision. The biomechanical model used in the present study relies on anthropometric based regression equations to estimate the hip joint centre [19]. The calculations are based on the distance between the anterior superior iliac spine and the greater trochanter, and the width of the pelvis (to define the medial-lateral position of the hip joint centre). If the hip joint centre estimation is incorrect it will affect the data derived from the analysis, not the least in individuals with achondroplasia [10]. This biomechanical model is still used in our lab, however, now with improved methods to track movement of the pelvis (i.e. by use of additional markers, and improved camera precision). There are other biomechanical models available that possibly could generate different results, particularly with regards to accuracy of defining the hip joint centre [24]. Furthermore, the number of statistical analyses performed in relation to the number of participants may increase the risk of type-1 errors. The strengths of the present study include the full description of gait characteristics including both kinematics, kinetics, and time and distance parameters, and an age-matched control group. Albeit 16 children may seem a limited study sample, it must be considered relatively large with respect to this patient population in a gait analysis context. Future research should focus on exploring the relationship between gait characteristics in children with achondroplasia and experienced physical symptoms, activity level, as well as well-being [9, 25]. It would also be of clinical importance to evaluate associations between gait patterns and functional mobility and capacity [26, 27].

## Conclusions

The findings from this cross-sectional study could serve as a baseline gait description for future studies evaluating gait in children with achondroplasia. Moreover, the results may be useful to clinicians involved in rehabilitation and can also be used to inform children and their families about gait characteristics in children with achondroplasia.

## Data Availability

The datasets analysed during the current study are not publicly available due to ethical concerns, but are available from the corresponding author on reasonable request.

## Abbreviations

3D: Three-dimensional
SD: Standard Deviation
N/A: Not Applicable
SH: Sitting Height
SHR: Sitting Height Ratio
SH SDS: Sitting Height Percent of Height in Standard Deviation Scores
